# Supplementary material for: Metagenomic binning with assembly graph embeddings
Source: Bioinformatics. 2022 Aug 16;38(19):4481–7. doi: 10.1093/bioinformatics/btac557 (PMC9525014; doi:10.1093/bioinformatics/btac557)
Supplement: btac557_supplementary_data [file btac557_supplementary_data.zip › amber outputs.zip!/amber outputs/strong100/index.html]

AMBER: Assessment of Metagenome BinnERs
